# Factors Influencing Total Delay of Breast Cancer in Northeast of China

**DOI:** 10.3389/fonc.2022.841438

**Published:** 2022-03-02

**Authors:** Sihang Ren, Yuting Zhang, Pan Qin, Jia Wang

**Affiliations:** ^1^ Department of Breast Surgery, Institute of Breast Disease, The Second Hospital of Dalian Medical University, Dalian, China; ^2^ Dalian No.3 People’s Hospital, Dalian Medical University, Dalian, China; ^3^ Faculty of Electronic Information and Electrical Engineering, Dalian University of Technology, Dalian, China

**Keywords:** breast cancer, total delay, survival analysis, logistic regression, individual–environmental–social factors

## Abstract

**Objectives:**

Delay in diagnosis and treatment, called total delay, could probably result in lower survival rates in breast cancer patients. This study aimed to investigate the factors associated with the comprehensive delay behaviors and to evaluate its effect on outcomes in patients with breast cancer in Dalian, a northeast city of China.

**Methods:**

A retrospective chart review was conducted using a cancer registry dataset including 298 patients. The Kaplan–Meier survival analysis was used to identify the threshold of total delay, dividing the patients into a group with significant uncertainty and a group without substantial delay. The factors associated with the significant total delay were investigated from the potential candidates, like income level and marital status, by using the chi-squared test. The difference of the clinicopathologic characteristics between the patients grouped by the significant total delay, like tumor size and lymph node metastasis, was also investigated to find out the effect of the total delay.

**Results:**

A total of 238 charts were used for analysis. The mean age was 57.3. The median of total delays was 3.75 months. Thirty days was identified as a threshold, more than which the total delay can lead to worse survival. Patients’ marital status (p = 0.010), income levels (p = 0.003), smoking status (p = 0.031), initial visiting hospital level (p = 0.005), self-health care (p = 0.001), and self-concern about initial symptom (p ≈ 0.000) were identified as the independent predictors of the total delay. Metastasis (p ≈ 0.000) was identified as the significant result relating to the significant total delay.

**Conclusions:**

A total delay of more than 30 days predicts worse survival in breast cancer patients in Dalian. Several factors, like patients’ marital status and income levels, can be considered to be relevant to the significant total delay. We recommend that these factors be used to predict the potential patients with the significant total delay in the clinical practice.

## Introduction

According to the National Central Cancer Registry of China, breast cancer is the dominant cause of cancer death in women younger than 45 years (1). Longer delays of diagnosis and initial therapy have been reported to result in cancer progression and poor survival (2, 3). This research investigated the socioeconomic factors and the clinical consequences associated with the total delay of breast cancer in Chinese women. The total delay includes two parts: 1) patient delay denotes the interval between the patient’s self-discovery of symptoms and the initial diagnosis; and 2) system delay denotes the interval between the initial diagnosis and the standard medical treatment. Patient delay always occurs in developing countries, and studies have reported that poor outcomes induced by patient delay are mainly due to low education and poor income status (4). Whether patient delay or system delay, some questions are pending, like the correct period of uncertainty and the possible associated factors that we could use to predict the delay cohort. Several works have investigated the associated delay factors for breast cancer patients, which show that the factors were diverse. Studies have shown that a lack of knowledge about breast cancer symptoms and screening methods is an essential factor in delaying diagnosis and seeking medical attention (5). The individual–environmental–social factors, like lower socioeconomic and recent immigration, were likely to delay medical help (6, 7). A study in South Africa found that most patients who delayed seeking help blamed poor transportation or treatment that interfered with work, dating, or even marriage (8). A cross-sectional study from China linked patient delay to perceived health competence (9). As shown by Khakbazan (10), some of the factors associated with the delay identified previously could not be generalized for different regions and races. For this reason, we collected the clinical data to infer the length of the total delay associated with the mortality, and we investigated the key factors influencing the total delay of breast cancer for women in the northeast of China.

## Materials and Methods

This cross-sectional study was conducted by data of patients diagnosed with breast cancers in the second hospital of Dalian Medical University between January 1, 2012, and December 31, 2012. Pathologists confirmed the diagnosis of breast cancer after surgery or core needle biopsy. Patients who did not complete standard adjuvant therapy were excluded. Patients with breast cancer were interviewed at the department of breast surgery after obtaining the agreement from each patient. We collected the information from the medical records of these patients. The follow-up questionnaire was conducted by phone call following the second year after patient diagnosis. All of the interviewers were previously trained residents not involved in the clinical management of the patients. We also excluded those patients who had no complete follow-up information. The record collection includes the following medical factors: initial symptom, family history of cancer, tumor molecular subtype, TNM stage, and metastasis. It also includes sociodemographic factors: age at presentation, marital status, marriage bonds, education level, residence, attitude to help-seeking, smoking habit, alcohol drinking patterns, insurance types, level of first visiting hospitals, occupation status, and self-health care. The results were analyzed using SPSS (version 25.0) and R language (version 3.5.1). We defined the significant total delay as the minimum delay leading to poor survival. The Kaplan–Meier survival analysis identifies the threshold for the significant total delay. The hypothesis test was used to distinguish the factors associated with the delay. Then, the label “1” was assigned to the patients with significant total delay and “0” to the patients without significant total delay. The multivariate logistic regression model was constructed concerning these labels to verify the factors identified using the hypothesis test.

## Results

### Study Population

A total of 296 charts were reviewed for this study. Fifty-seven charts did not meet inclusion criteria, and 238 charts were used for analysis. The sociodemographic characteristics of patients are illustrated in **Table 1**. In all patients, the mean age of patients was 57.3 ± 12.1 years. One hundred twenty-four (52.1%) patients resided in urban areas. One hundred two (42.4%) patients were single, where single included unmarried, divorced, and windowed. One hundred four (43.5%) patients were part-time employed, and 91 patients (38.1%) were full-time employed. One hundred sixty-four (62.8%) patients’ education levels were higher school or above. About the insurance status, 213 (89.1%) patients were with Medicare or Medicaid. One hundred eighteen (49.4%) patients were with no or low income. Fifty-five patients (22.2%) initially visited small local clinics. Ninety (37.7%) patients were self-concerned about the initial symptoms. Forty-six (19.2%) patients were smoking. Only 2 (0.8%) patients have an alcohol drinking habit. Forty-two (17.6%) patients conducted self-health care after discovering symptoms, like breast massage and taking traditional Chinese medicine. The medical history of patients is listed in **Table 2**. In all patients, the median of total delay was 3 months (0.1–12). Thirty-one (13.0%) died. Two hundred seven (87.0%) patients’ initial symptoms were lump, six (2.5%) patients’ initial symptoms were nipple changes, and fifteen (6.3%) patients’ initial symptoms were breast pain. Two hundred four (85.7%) patients have no family history of cancer. Metastasis happened in 75 (31.5%) patients. About the pathological types, triple-negative, HER2-enrich, Luminal A, and HR+HER2+ were diagnosed in 41 (17.2%), 12 (5.0%), 115 (48.1%), and 58 (24.3%), respectively. In all patients, the mean tumor size is 2.7 ± 1.8 cm. The detailed population of TNM classification can also be found in **Table 2**.

**Table 1:** Sociodemographic characteristics of patients (n = 238).

		N (%)
Age at presentation (years)	Mean ( ± SD)	57.3 ± 12.1
Area of residence	Rural	114 (47.9)
	Urban	124 (52.1)
Marital status	Married	137 (57.6)
	Single, divorced, or widowed	101 (42.4)
Occupation	No occupation	44 (18.5)
	Part-time employed	103 (43.3)
	Full time employed	91 (38.2)
Education level	Secondary or below	88 (37.0)
	Higher or above	150 (63.0)
Insurance	Self-pay	25 (10.5)
	Rural cooperative medical care	46 (19.3)
	Social insurance	167 (69.9)
Income level	No or low income	117 (49.2)
	Middle class or upper	121 (50.8)
Initial visiting hospital level	Small local clinic	55 (22.2)
	Large pandocheum	203 (77.8)
Self-concern about initial symptom	Yes	90 (37.8)
	No	148 (62.2)
Smoking	Yes	46 (19.3)
	No	192 (80.7)
Alcohol drinking habit	Yes	2 (0.8)
	No	236 (99.2)
Self-health care	Yes	42 (17.6)
	No	196 (82.4)

**Table 2:** Medical history of patients (n = 239).

		N (%)
Total delay (months)	Median [IQR]	3 [0.1, 12]
Vital status	Alive	207 (87.0)
	Dead	31 (13.0)
Initial symptom	Lump	208 (87.0)
	Nipple changes	7 (2.5)
	Breast pain	12 (6.3)
	Other	10 (4.2)
Family history of cancer	Yes	35 (14.6)
	No	203 (85.4)
Metastasis	Yes	75 (31.5)
	No	161 (67.6)
	Unknown	2 (0.8)
Pathological type	Triple-negative	41 (17.2)
	HER2-enrich	12 (5.0)
	Luminal A	115 (48.1)
	HR+HER2+	58 (24.3)
Tumor size	Mean ( ± SD)	2.7 ( ± 1.8)
TNM classification		
T	T0	1 (0.4)
	Tl	120 (50.4)
	T2	105 (44.1)
	T3	8 (3.3)
	T4	1 (0.4)
	Unknown	3 (1.3)
N	N0	117 (49.2)
	N1	112 (47.1)
	N2	6 (2.5)
	Unknown	3 (1.3)
M	M0	235 (98.7)
	M1	3 (1.3)

IQR, interquartile range.

### Total Delay of 30 Days Affecting Survival

**Figure 1:**
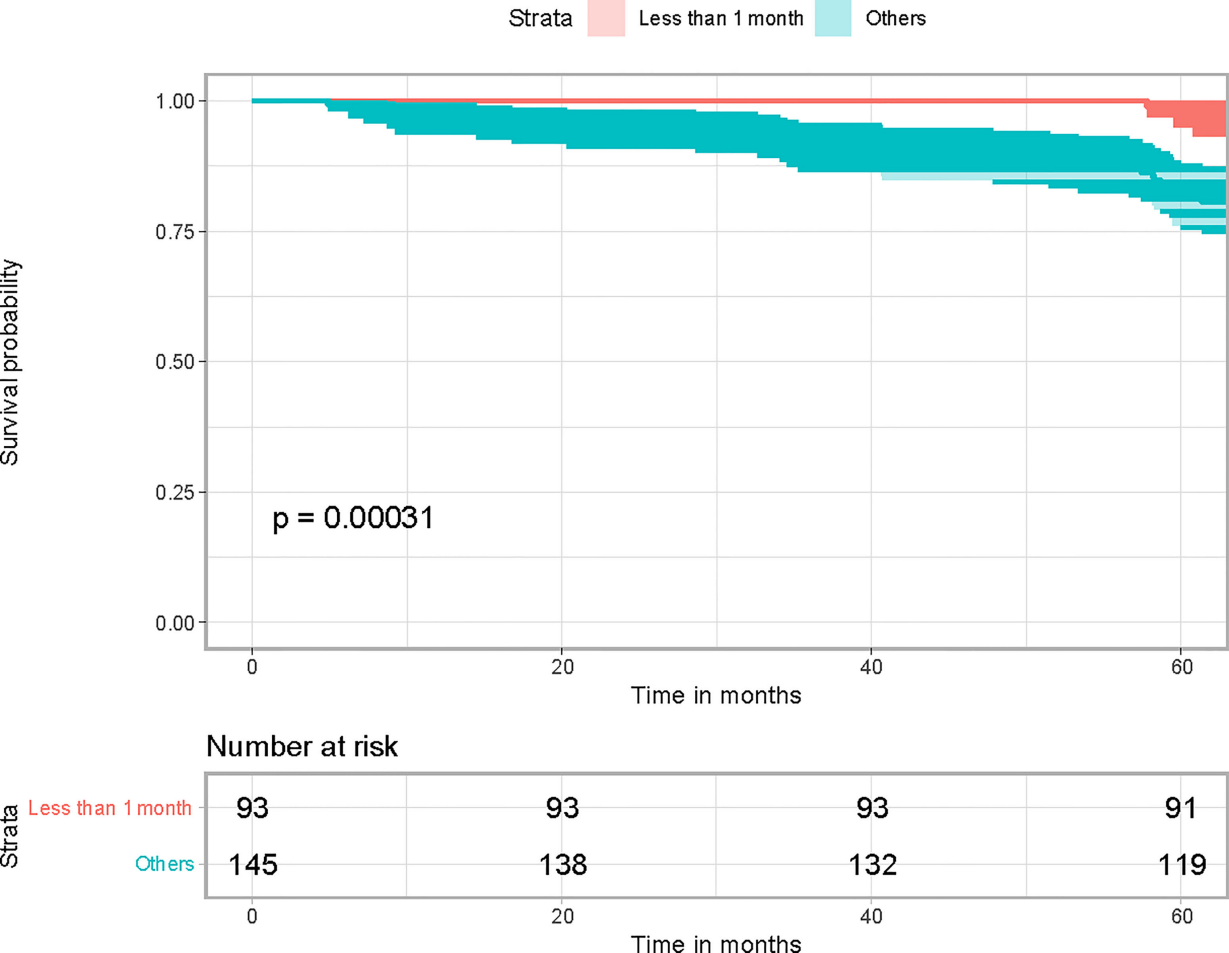
Kaplan–Meier survival analysis results.

We conducted the Kaplan–Meier survival analysis to investigate how long the total delay can affect the survival of patients. To test the significant difference in survival, we divided the patients into two groups by a threshold value of total delay. The threshold value was initially set as 10 days and was sequentially added by 10 days. We found that the total delay of 30 days can lead to significantly different survival curves (**Figure 1**). Therefore, we divided the patients into two groups: one group included the patients with total delays less than 30 days, and one group included the patients with total delays of more than 30 days.

### Significance Test for Grouped Patients

We also conducted the chi-squared test for the nominal variables to identify the significantly different factors for the grouped patients. Their marital status (p = 0.02), income levels (p = 0.003), smoking status (p = 0.03), insurance (p = 0.03), initial visiting hospital levels (p = 0.005), self-health care (p = 0.01), self-concern about initial symptom (p ≈ 0), and metastasis (p ≈ 0) were significantly different.

We consequently constructed the binomial logistic regression model by using the aforementioned significantly different variables as the covariates. The model has a percentage correction of 80.7%, which implies that significantly different factors can be used as the features for the classification of total delay.

## Discussion

Our study first found that the total delay of 30 days can lead to significantly different survival rates. The studies conducted in other countries and areas indicated that the diagnosis delay of cancers ranged from 2 to 15.2 weeks (11–16). Primarily, Ramirez evaluated 87 studies and suggested that the delay of 3 months could impact the long-term survival of breast cancer patients (11). Compared with this result, the delay in our investigated area was more pressing for the patients.

Various factors can prompt breast cancer patients to ignore their problems and delay medical treatment. The individual–environmental–social factors have been associated with the delay (17–19). According to the further application of the investigation results, we used the individual–environmental–social factors, including education level, occupation, income level, place of residence, particular dietary habit, smoking habit, alcohol drinking habit, insurance, marital status, insurance, first visiting the hospital, first consulted person, and self-health care.

According to our results, marital status was identified as an essential factor, not reported by other works. In our investigated area, the patients with no life partner tended to delay the medical diagnosis and treatment. Smoking was a relevant factor for 34 of 46 smoking patients who delayed seeking help. The smoking rate in our investigated cohort was 19.5%, much higher than the average level in China (the current smoking rate is 0.6%, and the ever-smoked rate is 3.4% among Chinese women) (20). Thus, the smoking status should be considered as an important factor in our investigated area.

Our results also indicated that patients with a low-income level were often associated with longer delays. This fact reflects the same panorama of other developing countries; e.g., Maghous and Fedewa confirmed that socioeconomic status appears to have a negative impact (4, 21).

We found that self-health care was a novel factor. Of 42 patients, 35 consequently conducting self-health care, like having a massage and acupuncture, have a longer delay. We suggested that self-health care could strengthen the patients’ self-confidence in their health status and become mindful of their symptoms. Atypical presenting symptoms of breast cancer lead to diagnostic intervals. To test this theory, we included the nature of breast masses and symptoms of breast disease. Although the results were not statistically significant, we believe that the study’s emphasis on a higher likelihood of delayed treatment for breast cancer patients without tumor symptoms is a reference (22).

Understanding and attention to initial symptoms and monitoring and managing symptoms have been the first and most crucial steps in the help-seeking process after symptom discovery (23, 24). In our study, the initial visiting hospital level and the patient’s self-concern about initial symptom were identified to be important factors, which are considered to have effects on the initial symptom interpretation and monitoring. The initial visiting hospital level was also identified as an essential factor associated with the delays. Forty of 52 patients who initially visited the small local clinic have delays. Women’s trust in the physicians’ professionalism was identified to affect patients’ help-seeking behavior (19, 25). This implied that the small clinics around Dalian city could not often offer proper initial symptom interpretation and monitoring. A total of 122 of 148 patients without self-concern about initial symptoms were identified to have delays. The reasons for negative attitudes toward the symptoms could be diverse. Economic status was the limiting factor for some patients. Some patients obtained the wrong symptom interpretation. The embarrassment of breast examination derived from traditional attitude could be the barriers to receiving care in Chinese middle-aged women (10).

Khokher stated that some of the factors associated with the delay identified previously could not be generalized for different races and regions (2). For example, Bleicher and Polverini have reported that the African American race was associated with delays in diagnosis and treatment (26, 27). However, the African American race often did not make up a higher percentage of Medicaid beneficiaries (28). Thus, rather than the race itself, the difference of economics between races can be considered a factor for the delay. However, such difference was not significant in Dalian, and as a result, ethics was not considered a potential factor.

To investigate what the delay will lead to, we also analyzed the difference between the delay and non-delay patients for the disease-associated factors, including the age of onset, initial symptom, family history of tumor, TNM stage, and molecular subtype. We found that 60 of 75 patients with metastasis have delays. This fact proved that the identified delay of 30 days could lead to advanced breast cancer.

As with other data analysis studies, this one has limitations. The data used in this research were collected from one large comprehensive hospital, which covered a quarter of patients in Dalian city. Our samples were not nationally representative due to its inclusion of patients seeking care at a single medical center. We did not group the negative attitudes to the symptoms, which could be directly related to other factors, like income level or self-health care. However, a similar study in Guangzhou, China, supports our research and shows that premenopausal patient status, breast disease history, and delayed physical examination affect the timing of patients’ visits (29).

Some studies show that the problem of delayed treatment is not very serious and that there is an ultimate delay time within which delayed treatment seems to be tolerated. Optimal times from diagnosis are <90 days for surgery, <120 days for chemotherapy, and, where chemotherapy is administered, <365 days for radiotherapy (30). The worldwide panic caused by COVID-19, the complication of medical procedures, and the difficulty of medical treatment for middle-aged and elderly patients have also primarily affected the enthusiasm of patients seeking medical treatment. However, as the influence of this period was not included in this study, no further details can be given (31).

In the future, we will also keep collecting data to testify and justify the statistical inference. We will propose an efficient prediction method for the patients’ delay status based on our identified factors. With the prediction method, we finally want to optimize the help-seeking behavior of the patients to shorten the delay.

## Data Availability Statement

The original contributions presented in the study are included in the article/supplementary material. Further inquiries can be directed to the corresponding authors.

## Ethics Statement

The studies involving human participants were reviewed and approved by the Second Affiliated Hospital of Dalian Medical University. The patients/participants provided their written informed consent to participate in this study.

## Author Contributions

SR, JW, and PQ wrote the manuscript and collected and cleaned the dataset. PQ and JW conducted the statistical analysis. SR, YZ, PQ, and JW edited the manuscript. All authors listed have made a substantial, direct, and intellectual contribution to the work and approved it for publication.

## Funding

This work was supported by the National Natural Science Research Foundation of China (81872247).

## Conflict of Interest

The authors declare that the research was conducted in the absence of any commercial or financial relationships that could be construed as a potential conflict of interest.

## Publisher’s Note

All claims expressed in this article are solely those of the authors and do not necessarily represent those of their affiliated organizations, or those of the publisher, the editors and the reviewers. Any product that may be evaluated in this article, or claim that may be made by its manufacturer, is not guaranteed or endorsed by the publisher.
